# Adapting Next-Generation Sequencing to *in Process* CRISPR-Cas9 Genome Editing of Recombinant *Ac*MNPV Vectors: From Shotgun to Tiled-Amplicon Sequencing

**DOI:** 10.3390/v17030437

**Published:** 2025-03-18

**Authors:** Madhuja Chakraborty, Lisa Nielsen, Delaney Nash, Jozef I. Nissimov, Trevor C. Charles, Marc G. Aucoin

**Affiliations:** 1 Department of Chemical Engineering, University of Waterloo, 200 University Avenue West, Waterloo, ON N2L 3G1, Canada; m6chakra@uwaterloo.ca (M.C.); lisa.beth.coles@uwaterloo.ca (L.N.); 2 Department of Biology, University of Waterloo, Waterloo, ON N2L 3G1, Canada; d2nash@uwaterloo.ca (D.N.); jnissimov@uwaterloo.ca (J.I.N.); tcharles@uwaterloo.ca (T.C.C.)

**Keywords:** CRISPR-Cas9, baculovirus, *Ac*MNPV, indel mutation, transfection-infection assay, next-generation sequencing, bioinformatics pipeline

## Abstract

The alphabaculovirus *Autographa californica* multiple nucleopolyhedrovirus (*Ac*MNPV) is the most commonly used virus in the Baculovirus Expression Vector System (BEVS) and has been utilized for the production of many human and veterinary biologics. *Ac*MNPV has a large dsDNA genome that remains understudied, and relatively unmodified from the wild-type, especially considering how extensively utilized it is as an expression vector. Previously, our group utilized CRISPR-Cas9 genome engineering that revealed phenotypic changes when baculovirus genes are targeted using either co-expressed sgRNA or transfected sgRNA into a stable insect cell line that produced the Cas9 protein. Here, we describe a pipeline to sequence the recombinant *Ac*MNPV expression vectors using shotgun sequencing, provide a set of primers for tiled-amplicon sequencing, show that untargeted baculovirus vector genomes remain relatively unchanged when amplified in Sf9-Cas9 cells, and confirm that *Ac*MNPV *gp64* gene disruption can minimize baculovirus contamination in cell cultures. Our findings provide a robust baseline for analyzing *in process* genome editing of baculoviruses.

## 1. Introduction

Baculovirus vectors are versatile tools for producing recombinant products in insect cells. Together, they are commonly referred to as the Baculovirus Expression Vector System (BEVS). The BEVS platform utilizes a recombinant *Autographa californica* multiple nucleopolyhedrovirus (*Ac*MNPV) baculovirus that can infect cultured cells derived from the ovarian tissue of its natural hosts, such as the Fall Armyworm (*Spodoptera frugiperda*; Sf21 or Sf9 cells) and the Cabbage Looper (*Trichoplusia ni*; High-Five^TM^ or Hi-5 cells) [1]. The double-stranded DNA genome of the *Ac*MNPV C6 strain is around 134 kbp and contains ~155 open reading frames (ORFs) or genes [2,3,4]. Although BEVS has gained popularity in laboratory settings, its application in industry remains limited. A bottleneck in the improvement of this system is that the functions of many *Ac*MNPV genes have yet to be experimentally determined. In fact, most of the commercially available *Ac*MNPV DNA backbones remain relatively unchanged [5]. However, improvements in recombinant protein production and genome stability have been achieved by removing certain *Ac*MNPV genes [6,7,8]. This shows that the BEVS platform could benefit from a systemic screening of genes to identify their essentiality. The removal or disruption of genes not essential for the production of foreign proteins in cell culture has the potential to increase the production of foreign proteins [9]. Using CRISPR-Cas9 gene editing technology, baculovirus genes can be targeted in the production phase without the need for gene-specific complementary cell lines.

CRISPR-Cas9 is a highly efficient tool for targeted *in vivo* and *in vitro* genetic engineering. Of *Drosophila melanogaster* S2 cells that gained resistance to puromycin from a plasmid that also contained the *cas9* gene and single guide RNA (sgRNA), 88% of the alleles were found to have indel mutations [10]. CRISPR has also been used to identify genes essential for *Bombyx mori* BmE cell viability through a genome-wide screen [11]. CRISPR-Cas9 genome editing in insect antiviral research was first reported in 2016 and it showed that an inducible CRISPR-Cas9 system (p39 K-Cas9-sgRNA-mCherry), activated upon viral infection, could reduce the possibility of off-targets and produce transgenic silkworms resistant to *Bombyx mori* nucleopolyhedrovirus (*Bm*NPV) [12]. Two other studies constructed inducible CRISPR-Cas9 systems to introduce antiviral activity in transgenic silkworms by utilizing the *Bombyx mori* U6 promoter for expression of sgRNAs and the *Bm*NPV 39k promoter for Cas9 expression only upon viral infection [13,14]. One study created a transgenic line expressing Cas9 and sgRNA to knock out the host *BmATAD3A* gene and inhibit *Bm*NPV replication [13], while the other used four sgRNAs targeting the *Bm*NPV genes essential for viral replication, thus providing the transgenic silkworm with resistance to *Bm*NPV infection [14].

U6 promoters with transcriptional activity for sgRNA expression in *S. frugiperda* and *T. ni* were not reported on until 2017 [15]. This led to a study by Bruder et al. in 2021 that implemented the CRISPR-Cas9 tools in the BEVS, specifically the baculovirus *Ac*MNPV and Sf9 insect cells [16]. This work utilized engineered Sf9 cell lines expressing *cas9* or *dcas9* to compare targeted gene disruption (CRISPRd) or transcriptional repression (CRISPRi), respectively [16]. Building on this previous work, rBEVs with the reporter gene replaced with the human immunodeficiency virus type 1 (HIV-1) *gag* gene, also carrying sgRNAs targeting the *Ac*MNPV *gp64* or *vp80* genes, were constructed to reduce baculovirus co-production in cell cultures [17]. CRISPR-mediated disruption of the *gp64* or *vp80* genes resulted in ~99% or ~94% reduction in infectious virus titers (IVTs), respectively, without impacting Gag virus-like particle (VLP) production [17]. An alternative strategy to minimize baculovirus contamination utilized an inducible CRISPR-Cas9 knockout system based on co-infection with two rBEVs carrying either the *cas9* endonuclease or a sgRNA for the *gp64* or *vp80* genes [18]. While knocking out these genes resulted in a more than 90% reduction in virus titer, it also reduced the overall fluorescence intensity of the enhanced yellow fluorescent protein (eYFP) acting as a surrogate for recombinant protein production [18]. Another study developed a CRISPR-Cas9 based transfection-infection assay to effectively scrutinize the baculovirus genes for essentiality by transfecting Sf9-Cas9 cells with sgRNA plasmid followed by infecting with a rBEV carrying green fluorescent protein (GFP) and evaluate its impact on foreign protein and IVT production [5].

Baculoviruses are prone to mutations during replication processes and inherently form defective interfering particles upon propagation in insect cell cultures [3,8], thus making genome sequencing an important tool to investigate the baculovirus population being employed for various applications including, but not limited to, biologics production. Although phenotypic confirmation of CRISPR-Cas9 activity has previously been reported [5,16,17], an overall look at the entire rBEV genome upon targeted gene disruption has yet to be thoroughly examined. In addition, a deep understanding of the rBEV sequence is required prior to designing sgRNAs to target different genes of the rBEV. This paper addresses some of the current shortcomings and details the methodologies required to achieve these tasks.

In this work, we initially use shotgun sequencing on infected cell culture supernatant and describe the pipeline to generate a consensus sequence of the rBEV. While shotgun sequencing allows sequencing of unknown samples, it requires large quantities of DNA input; has the potential to introduce assembly errors, especially around repetitive regions; and would require additional processing to sequence intracellular baculovirus genomes. Thus, we decided to use a whole-genome tiled-amplicon sequencing assay that is robust, requires less DNA, provides high coverage, and is specific to genome(s) of interest [19]. We adapted this tiled-amplicon approach [19] for the rBEV genomes recovered from the multi-well plate experiments, which uses Sf9-Cas9 cells as the host of baculovirus infection, and reported on the whole-genome sequences of these targeted and untargeted rBEV gDNAs (major species) and confirmed the targeted mutations. While the consensus sequence of the rBEV enabled us to confidently design sgRNAs to target specific *Ac*MNPV genes, the more specific tiled-amplicon sequencing assay provided a foundation to study the whole *in process* rBEV genome and confirm the mutations upon CRISPR-Cas9 mediated gene targeting. This is especially important if you want to target genes whose disruption can reduce baculovirus contamination and facilitate the purification process of your products, such as recombinant proteins or antigens for vaccines.

## 2. Materials and Methods

### 2.1. Cell Line and Maintenance

Parental Sf9 cells and Cas9 expressing Sf9 cells (Sf9-Cas9, consisting of a Cas9-2A-puromycinR gene cassette) [16] were maintained in capped glass Erlenmeyer flasks in Sf-900^TM^ III serum-free media (SFM) (Gibco, Carlsbad, CA, USA) at 27 °C and 130 rpm. The cells were passaged every 3–4 days when the viable cell density reached between 3 × 10^6^ cells/mL and 5 × 10^6^ cells/mL. Additionally, puromycin (Sigma-Aldrich, Oakville, ON, Canada) was added to the Sf9-Cas9 cells every other passage at a concentration of 5 µg/mL to ensure *cas9* expression.

### 2.2. Baculovirus Amplification and Quantification

Two recombinant baculoviruses were used in this work: a recombinant baculovirus expressing the GFP monomeric Azami green (mAG) under the *Ac*MNPV late basic p6.9 promoter [5], herein referred to as p6.9GFP rBEV; and a baculovirus that co-expresses a sgRNA under a SfU6 promoter and mAG under p6.9 promoter [17], herein referred to as p6.9GFP_sgRNA_gp64+131 rBEV. The baculoviruses were amplified in Sf9 cells at a low MOI (~0.1 pfu/cell) until cell viability dropped between 80 and 90% to obtain a working virus stock for each rBEV.

Infectious virus titers were quantified by end-point dilution assay (EPDA), as previously described [20,21]. Briefly, each well of 96-well tissue culture-treated plates (VWR International, Mississauga, ON, Canada) was seeded with 100 µL of Sf9 cells diluted to 2 × 10^5^ cells/mL and allowed to adhere for around 1 h at 27 °C. During the incubation period, virus stocks were serially diluted from 10^−2^ to 10^−8^ using Sf-900^TM^ III SFM. The cells were then inoculated with 10 µL of a virus dilution resulting in 12 replicates per dilution. The plates were incubated at 27 °C for 6–7 days and observed under a fluorescence microscope to determine green fluorescence. The reciprocal of TCID_50_ expressed in mL of virus added (0.01 mL) was multiplied by 0.68 based on the Poisson distribution to obtain the virus titer in plaque-forming units per mL (pfu/mL).

### 2.3. Plasmid Design and Construction

All primers used in this study were synthesized by Integrated DNA Technologies (IDT) (Coralville, IA, USA) and can be found in Supplementary Table S1. A previously constructed sgRNA plasmid with a scrambled spacer sequence was used as the control plasmid in this work [22]. Briefly, a PCR-amplified SfU6 promoter [15] gBlock gene fragment and gRNA scaffold along with a transcriptional terminator (Addgene # 49411) [23] were amplified in a fusion PCR reaction to obtain the SfU6-sgRNA insert with a scrambled spacer sequence (5′-3′ caccttgaagcgcatgaact). The backbone containing the ampicillin resistance gene (ampR) and the pBR322 origin of replication (ori) from the pBR322-TIMER plasmid (Addgene # 103056) [24] was PCR-amplified separately to obtain the backbone fragment. Finally, the SfU6-sgRNA insert and the backbone fragment were assembled in a Gibson assembly reaction to construct the pSfU6-sgRNA scrambled control plasmid. Additionally, the gp64+131 plasmid with a *gp64* targeting spacer sequence (5′-3′ ggaaacgctgcaaaaggacg) at the +131 location from the first nt within *gp64* was obtained from a previous study [5].

### 2.4. Shotgun Sequencing of the p6.9GFP rBEV

In this study, shotgun sequencing was used to sequence the genome of the p6.9GFP rBEV recovered from the supernatant of the infected Sf9 cells in suspension. Next, 15 mL of virus stock was filtered with a 0.22 µm PES filter and the gDNA was extracted using the AllPrep PowerViral DNA/RNA kit (Qiagen, Hilden, Germany) according to the manufacturer’s protocol without bead beating. To obtain enough starting material for sequencing sample preparation, multiple gDNA extractions were performed and the pooled extracts were concentrated with AMPure XP magnetic beads (Beckman Coulter, Mississauga, ON, Canada). The gDNA bound to the beads was eluted in a smaller volume to increase the starting material concentration, thus keeping the DNA yields high throughout the sample preparation steps. Using a dsFragmentase enzyme, gDNA was fragmented for short-read sequencing. The fragmented gDNA was then prepared for shotgun sequencing with the NEBNext^®^ Ultra^TM^ II DNA Library Prep Kit for Illumina^®^ (New England Biolabs, Whitby, ON, Canada) according to the manufacturer’s instructions. Briefly, end-repair, 5′ phosphorylation, and dA-tailing were performed on the fragmented gDNA. This was followed by the ligation of bell-shaped adapters to the fragments and then cleavage of the bell-shape by U-excision. The fragments were cleaned and size-selected, and PCR enrichment was used to add Illumina barcodes and sequencing adapters to the fragment ends. The prepared DNA library was sequenced on the MiSeq platform using 2 × 250 sequencing with a MiSeq reagent kit v2 (Illumina, San Diego, CA, USA).

A best-guess sequence of the p6.9GFP rBEV, generated by combining the *Ac*MNPV DNA backbone and a transfer vector carrying the GFP gene [5], was used as the reference genome to assemble our working viral p6.9GFP rBEV genome. Sequenced raw reads were processed with Trimmomatic [25] to remove low-quality reads and index sequences. The short Illumina reads were then aligned to the reference genome of the p6.9GFP rBEV using BBtools [26] to produce a sam file of mapped, partially mapped, and unmapped reads. The sam file was converted to a bam file using samtools [27] and Anvi’o [28]. The bam file was then polished with Pilon [29] to resolve differences such as insertions, deletions, and single-nucleotide polymorphisms (SNPs) between the reads and the reference genome, which produced a FASTA file containing an improved representation of our working rBEV genome. Finally, using the MAFFT-7.0 alignment algorithm [30], the reference genome and the newly assembled genome were aligned and the DNAdiff tool was used to identify differences and similarities between the two. The sequence of the newly assembled genome (hereby referred to as the shotgun-sequenced or working rBEV) was then used for all future work involving the p6.9GFP rBEV.

### 2.5. Transfection in Multi-Well Plates

A previously developed CRISPR-Cas9 based transfection-infection assay (T-I assay) was used in this study [5]. Briefly, Sf9-Cas9 cells were seeded on a tissue-culture-treated 12-well plate (VWR International, Mississauga, ON, Canada) at a density of 0.9 × 10^6^ cells/well and allowed to adhere for around one hour at 27 °C. The cells were then transfected with the sgRNA scrambled control plasmid or the gp64+131 plasmid using FuGENE HD (Promega, Madison, WI, USA) transfection reagent following the manufacturer’s protocol.

### 2.6. Infection in Multi-Well Plates

For experiments conducted using the p6.9GFP_sgRNA_gp64+131 rBEV, Sf9-Cas9 cells were seeded on a tissue culture-treated 12-well plate (VWR International, Mississauga, ON, Canada) at a density of 0.9 × 10^6^ cells/well and incubated overnight at 27 °C. The following day, the media were replaced with fresh Sf-900^TM^ III media containing the p6.9GFP_sgRNA_gp64+131 rBEV or the p6.9GFP rBEV (infected-only control) to achieve synchronous infection. For experiments involving transfection, 16–24 h post-transfection (hpt), the media were aspirated from each transfected well, and fresh Sf-900^TM^ III SFM containing p6.9GFP rBEV was added to achieve an infection at an MOI of 3 pfu/cell.

### 2.7. Harvesting from Multi-Well Plates

Approximately 48 h post-infection (hpi), the cell cultures were harvested by centrifugation at 800× *g* for 15 min and the cell pellets were analyzed by flow cytometry. The supernatants were stored at 4 °C for further analysis by EPDA and next-generation sequencing (NGS) and the cell pellets treated with the p6.9GFP_sgRNA_gp64+131 rBEV or the gp64+131 plasmid were stored at −80 °C for NGS analysis.

### 2.8. Flow Cytometry Analysis

Cell pellets were resuspended in 2% paraformaldehyde in phosphate-buffered saline (PBS) followed by incubation at 4 °C for 30 min. The samples were then diluted in 1× PBS and analyzed by a BD Accuri^TM^ C6 Plus flow cytometer (BD Biosciences, San Jose, CA, USA) equipped with a 488 nm excitation frequency blue laser. Samples were run at a low flow rate (14 µL/min) and 10,000 events were collected for each sample. The acquired flow data were analyzed by FlowJo^TM^ V10 software (Tree Star, Ashland, OR, USA). After applying gates to remove cell debris and intrinsic cellular fluorescence, the geometric mean fluorescence intensity captured by the FL1 detector was calculated. The R programming language was then used for downstream data analysis and visualization.

### 2.9. Tiled-Amplicon Sequencing Assay for rBEV Genomes

In this work, the tiled-amplicon sequencing assay was used to sequence the rBEV gDNAs recovered from multi-well plates. This whole genome NGS assay was adapted from the paper ‘Multiplex PCR method for MinION and Illumina sequencing of Zika and other virus genomes directly from clinical samples’ [19] and it uses a set of target-specific primers to amplify a genome. Primer sets bind across the genome to PCR-amplify specific regions, called amplicons, that make up the entire genome. Each successive primer pair overlaps with the next; thus, the overlapping PCR amplicons can be assembled to generate the entire genome.

#### 2.9.1. Primer Design and Multiplex PCR

**Figure 1:**
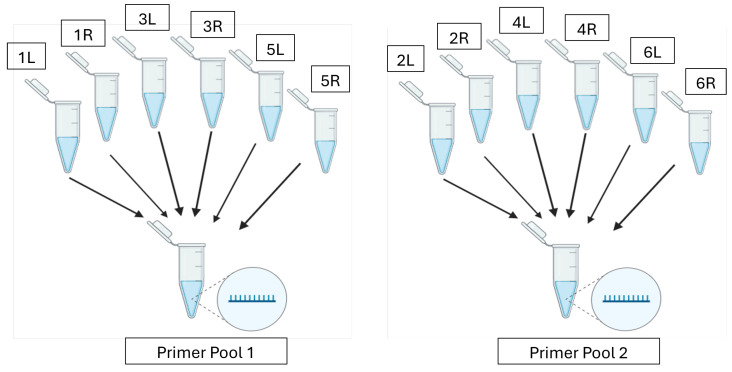
Odd-numbered forward (L) and reverse (R) primers are combined to make Primer Pool 1 and even-numbered forward (L) and reverse (R) primers are combined to make Primer Pool 2.

PrimalScheme [19] was used to design primers to amplify 5000 nt tiled segments of the rBEV gDNA extracted using the Wizard Genomic DNA Purification kit (Promega, Madison, WI, USA) following the manufacturer’s protocol. To design the primer scheme, the FASTA file of the shotgun-sequenced rBEV was selected as the reference genome and the amplicon length was set to 5000 nt. PrimalScheme recognized the input genome as linear; thus, to ensure 100% coverage of the circular genome, an additional amplicon that overlapped with the first and last amplicons was used to close the gap. Briefly, the first 3000 nt of the genome was added at the end of the last 2300 nt and it was run through PrimalScheme while setting the amplicon length to 5000 nt. Upon running the two primer schemes, the program returned files containing the primer locations and primer pairs (Supplementary Tables S2 and S3, respectively) which were subsequently ordered from IDT. The lyophilized tiled-amplicon primers were resuspended in nuclease-free water using the volumes suggested by IDT to achieve a stock concentration of 100 µM. Primer pools were then prepared by pooling primer pairs producing odd-numbered amplicons (Primer Pool 1) separately from those producing even-numbered amplicons (Primer Pool 2) to a total concentration of 10.2 µM for each pool and 0.34 µM for each primer (Figure 1). This ensured primer pairs for alternate regions were pooled together and the amplicons overlapped between the pools. The tiled-amplicon primers generated for the shotgun-sequenced rBEV were utilized for all tiled-amplicon sequencing assays in this study.

A multiplex PCR method generated 5000 nt amplicons with a short ~75 nt overlap between each PCR amplicon. This method allowed amplification of the whole genome with alternate rBEV genome regions amplified by Pool 1 or Pool 2 primers [19]. Two PCR reactions (PCR Pool 1 and PCR Pool 2) were set up for each sample. The multiplex PCR setup and amplification parameters are shown in Table 1 and Table 2, respectively.

#### 2.9.2. DNA Amplicon Cleanup and Quantification

First, 2 µL of the PCR products was run on 1% agarose gel to confirm the presence of 5000 nt amplicons and visually compare the Pool 1 and Pool 2 band intensities for each sample. Following that, Pool 1 and Pool 2 PCR products of a sample containing a similar amount of the 5000 nt amplicons were combined in a 96-well PCR plate. Next, 32 µL of room temperature AMPure XP magnetic beads (Beckman Coulter, Mississauga, ON, Canada) was added to the combined 40 µL PCR products for each sample and cleanup was performed according to the manufacturer’s protocol. Finally, 20 µL of purified DNA amplicons, eluted in 22.5 µL Sox 5 (10 mM Tris-HCl, pH 8.5), was transferred to new wells in the PCR plate.

Quantification of 2 µL of the purified amplicons was performed using the Qubit dsDNA high-sensitivity assay kit (Thermo Fisher Scientific, Waltham, MA, USA) with a Qubit 4 Fluorometer (Invitrogen by Thermo Fisher Scientific) in accordance with the manufacturer’s instructions. All the concentrations were recorded for further sample preparation.

#### 2.9.3. DNA Library Construction

Custom index and adapter primers were synthesized by IDT (Supplementary Table S4) and were diluted to 10 µM stocks stored at −20 °C. Illumina DNA Prep, (M) Tagmentation kit (Illumina, San Diego, CA, USA) was used to prepare library DNA for sequencing according to the manufacturer’s protocol. Briefly, bead-linked transposomes (BLTs) were used for DNA tagmentation, in which the DNA were fragmented and tagged with adapters (5′-3′ CTGTCTCTTATACACATCT). This was followed by a cleanup step that involved stopping the tagmentation process and washing the adapter-tagged DNA. The tagmented DNA was then amplified using a reduced-cycle PCR that added index 1 (i7) adapters, index 2 (i5) adapters, and sequences necessary to generate sequencing-ready DNA fragments. Finally, the DNA library amplicons were purified using a double-sided bead purification process that excluded index primers and primer dimers. A Qubit dsDNA high-sensitivity assay kit (Thermo Fisher Scientific, Waltham, MA, USA) with a Qubit 4 Fluorometer (Invitrogen by Thermo Fisher Scientific) was then used to quantify the indexed amplicons as described by the manufacturer and median sizes of each DNA library (400 nt) were estimated by running them on 1% agarose gel.

#### 2.9.4. Sequencing DNA Library Preparation

The concentration of the indexed amplicons in ng/µL was converted to nM using Equation (1) for sequencing cluster generation and each DNA library was diluted to 4 nM in Sox 5 solution for sequencing preparation.

(1) $$concentration\;in\;\mathrm{nM}=\frac{concentration\;in\;\mathrm{ng}/µL\;}{660\;g/\mathrm{mol}\times average\;library\;size\;in\;\mathrm{bp}}$$

Each 4 nM DNA library was denatured using a freshly made 0.2 N NaOH solution. After 5 min of incubation at room temperature, the reaction was stopped using pre-chilled hybridization buffer (HT1) to obtain 1 mL of 20 pM denatured DNA library. Using HT1, the 20 pM denatured DNA library was diluted to the desired concentration. The 10 nM PhiX sequencing control (Illumina, San Diego, CA, USA) was also diluted to 4 nM in Sox 5 solution and the 4 nM PhiX library was then denatured and diluted as the DNA libraries. Finally, 588 µL of denatured DNA library was combined with 12 µL of denatured PhiX library and loaded onto the MiSeq reagent kit v3 (Illumina, San Diego, CA, USA) cartridge for sequencing.

### 2.10. Bioinformatics Pipeline for Major Species

**Figure 2:**
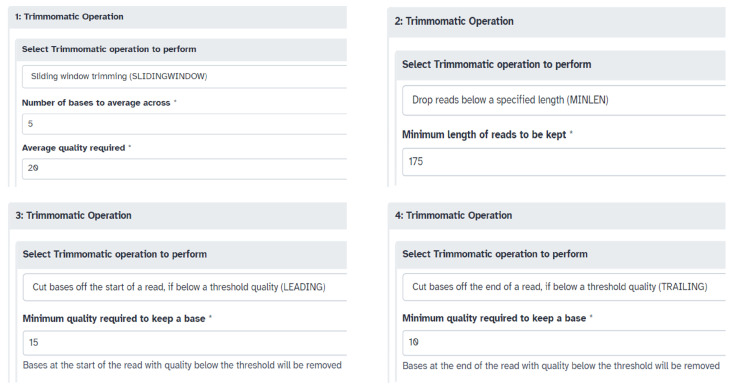
Parameters used for the four Trimmomatic operations. The * here indicates a mandatory field.

Recombinant baculovirus tiled-amplicon sequencing reads were processed and analyzed using the Galaxy online platform (https://usegalaxy.org/, accessed on 28 November 2023). Reference genome(s), Illumina adapter sequences, and forward (R1) and reverse (R2) raw read files were uploaded to the server, followed by an assessment of read quality performed using the FastQC tool (Galaxy Version 0.74+galaxy1). The Trimmomatic tool (Galaxy Version 0.39+galaxy2) was then used to trim the paired-end reads by selecting the ILLUMINACLIP step for custom adapter removal and performing 4 Trimmomatic Operations (SLIDINGWINDOW: 5:20, MINLEN: 175, LEADING: 15, TRAILING: 10) (Figure 2). The parameters of the Trimmomatic Operations can be changed if needed. Using FastQC, the quality of trimmed paired reads was analyzed and the files passed the adapter content criteria. The BWA-MEM2 tool (Galaxy Version 2.2.1+galaxy1) was used to build an index and map the forward and reverse trimmed paired reads to a genome from its history (reference genome). Optionally, samtools depth (Galaxy Version 1.15.1+galaxy2) could be run on the bam file to obtain the read depth at each position. An automated genome assembly tool, Pilon (Galaxy Version 1.20.1), then aligned the bam file (BWA-MEM2 alignment file) of one sample at a time with the reference genome to generate a Pilon-assembled genome. Following this, the DNAdiff tool (Galaxy Version 4.0.0+galaxy1) was used to evaluate similarities and differences between the Pilon assembled genome of each sample and the reference genome. Lastly, all the FASTA files were downloaded and pasted into a single file for visualization of genome alignments and identification of indel mutations performed by the MAFFT version 7 server (https://mafft.cbrc.jp/alignment/server/, accessed on 28 November 2023) [30].

To further confirm the CRISPR-Cas9 mediated mutations within the targeted *gp64* gene, the Galaxy online platform was run from a different server (https://usegalaxy.be/, accessed on 15 February 2025) that provided a tool to analyze CRISPR-based mutations specifically. Briefly, after assessing the quality of the reads by FastQC, the Trimmomatic tool was used to remove adapters and low-quality reads (ILLUMINACLIP with custom adapters, SLIDINGWINDOW: 4:20, MINLEN: 50, LEADING: 15, TRAILING: 10). Following this, FastQC was performed on the trimmed paired reads to ensure adapter removal and quality. Finally, the CRISPResso2 tool (Galaxy Version 0.1.1) [31,32] was run utilizing the trimmed paired-end reads, a *gp64* amplicon sequence (the first 238 nucleotides of the *gp64* gene), the gp64+131 sgRNA sequence, and the following parameters—flexiguide homology: 60, minimum overlap length between reads: 5, maximum overlap length between reads: 251, and center of quantification window to use with respect to the 3′ end of the provided sgRNA sequence: −3.

## 3. Results

### 3.1. Consensus Sequence of p6.9GFP rBEV from Shotgun Sequencing

The *Ac*MNPV C6 strain sequence (NCBI accession number: NC_001623.1) [2], modified to accommodate changes for use as an rBEV (the *chiA* and *v-cath* genes were removed and the promoter and reporter gene, i.e., p6.9GFP, were added) was used as a reference genome. To confirm the rBEV sequence prior to targeting different genes in the virus vector, shotgun sequencing was performed. Shotgun sequencing was selected for whole-genome sequencing of the p6.9GFP rBEV because it does not require specific knowledge of the DNA sequence. Twelve million raw reads were obtained, and around eight million processed reads passed the quality filters. The polished sequencing data were manually annotated and provided a better understanding of the genome by highlighting key differences between the assumed and the actual sequences of the rBEV. The shotgun-sequenced rBEV (131,545 bp) was ~400 bp shorter than the reference genome (131,944 bp) and included 15 insertions, 11 deletions, and 52 SNPs in various regions of the genome (Table 3). Among the mutations, a 37 bp deletion and a 7 bp SNP were seen in the 45 bp region between where *chiA* and *v-cath* genes were originally located. This region potentially served as the promoter for the bi-directional adjacent *chiA* and *v-cath* genes. Moreover, the homologous repeat regions (hrs) had five indel mutations ranging from 1 bp to 137 bp and seven SNPs of 1 bp each. While the *Ac*MNPV genome has 9 hrs in different locations; specifically, hr1, hr1a, hr2, hr2a, hr3, hr4a, hr4b, hr4c, and hr5; the hrs mutations were confined to hr2, hr3, and hr5, as shown in Table 3. The repetitive nature of the hrs could lead to sequencing or mapping errors and could be mutation hotspots [33]. The remaining mutations could be attributed to errors in the reference genome sequence [34] or differences in sequence properties of the original *Ac*MNPV C6 strain [2] and our rBEV stocks. A FASTA file of the shotgun-sequenced rBEV has been deposited with Borealis, The Canadian Dataverse Repository (https://doi.org/10.5683/SP3/FIBEX4, accessed on 20 February 2025). The shotgun-sequenced rBEV was used to design primers for tiled-amplicon sequencing and any sgRNA designed using the online tool CHOPCHOP (https://chopchop.cbu.uib.no/, accessed on 22 July 2023) [35] was matched with the sequenced genome to confirm the target locations.

### 3.2. Tiled-Amplicon Sequencing of p6.9GFP rBEV

Extracted p6.9GFP rBEV gDNA from the multi-well plate’s infected-only control was sequenced using the tiled-amplicon sequencing assay, utilizing the shotgun-sequenced rBEV as the reference genome. This assay overcame some of the challenges faced with shotgun sequencing by being more specific and requiring a lower concentration of input DNA. A total of 475,872 read pairs were obtained and 90.97% of them passed the quality control, and 99.75% of the filtered reads aligned with the reference genome. The reads covered 100% of the genome with an average depth of 2006.12×. This confirmed that the designed tiled-amplicon primers covered the entire rBEV genome and could be used for future sequencing work. Compared to the reference genome, the more specific tiled-amplicon sequencing revealed a 35 bp deletion in the hr2 (aaatgatgtcattggatgagtcatttgtttttcaa). A visual representation of sequence alignment compared to the reference genome can be found in Supplementary Figure S1. The FASTA file of the tiled-amplicon-sequenced p6.9GFP rBEV from the infected-only control has been deposited in Borealis, The Canadian Dataverse Repository (https://doi.org/10.5683/SP3/FIBEX4, accessed on 20 February 2025).

### 3.3. Lowering Infectious Baculovirus from Sf9-Cas9 Cells Through Co-Expressing a sgRNA Targeting *gp64*

**Figure 3:**
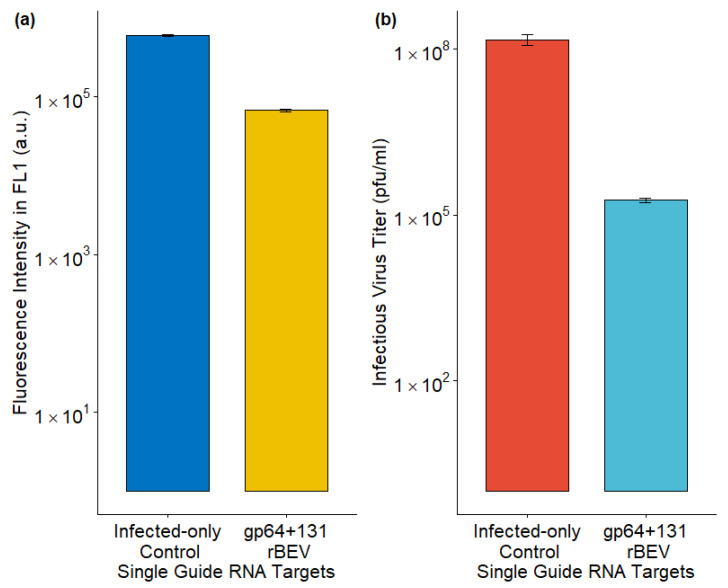
Impact of sgRNA rBEV-mediated *gp64* gene disruption on (**a**) foreign protein production and (**b**) infectious virus titer. The data from 3 replicates are presented here.

Sf9-Cas9 cells were infected with the p6.9GFP_sgRNA_gp64+131 rBEV for targeted *gp64* gene disruption. Sf9-Cas9 cells infected with p6.9GFP rBEV served as the control here. Although the rBEVs are slightly different, they carry the same foreign protein, thus enabling a comparison between the targeted and untargeted rBEVs. Using the p6.9GFP_sgRNA_gp64+131 rBEV, the IVT was significantly reduced compared to the control (Figure 3b). On the other hand, foreign protein production, as measured by fluorescence intensity, was only slightly lower compared to the control (Figure 3a). These data indicate that disrupting *gp64* could minimize budded virus co-production in cell cultures.

The gDNA of the p6.9GFP_sgRNA_gp64+131 rBEV amplified in Sf9 cells (where no Cas9 was present) was sequenced by tiled-amplicon sequencing and used as the reference genome for analyzing mutations following the infection of Sf9-Cas9 cells with this rBEV. After *gp64* gene disruption, rBEV gDNA could only be recovered from the cell pellet fraction, thus emphasizing the need for tiled-amplicon sequencing to amplify the specific intracellular genome of interest. Compared to the reference genome, the consensus sequence of the p6.9GFP_sgRNA_gp64+131 rBEV following *gp64* targeting exhibited six SNPs and two indels in various regions of the gDNA (Table 4). The SNPs in hr3, *AcOrf-11*, and *AcOrf-84* and the two indel mutations within *pk-1* were also observed in the shotgun-sequenced rBEV. However, the CRISPResso2 sequencing pipeline was not able to detect any mutation within the *gp64* gene, even though phenotypic changes were observed. The FASTA files of the untargeted and targeted p6.9GFP_sgRNA_gp64+131 rBEV, and the detailed report generated by CRISPResso2 for the targeted rBEV can be accessed via Borealis, The Canadian Dataverse Repository (https://doi.org/10.5683/SP3/FIBEX4, accessed on 20 February 2025).

### 3.4. Tiled-Amplicon Sequencing of p6.9GFP rBEV upon T-I Assay

The extracted p6.9GFP rBEV gDNA from the multi-well plate’s scrambled control (cells transfected with a plasmid coding for a random sgRNA that did not target any baculovirus gene) was sequenced using the tiled-amplicon sequencing assay and compared to the shotgun-sequenced rBEV, which was used as the reference genome. A total of 966,391 read pairs were obtained, with 94.59% of the reads passing the quality filter thresholds while 99.65% of those reads aligned with the reference genome. The reads covered 100% of the genome with an average depth of 4063.722×. Compared to the reference genome, the tiled-amplicon sequencing revealed a 35 bp deletion in the hr2 (aaatgatgtcattggatgagtcatttgtttttcaa), a 20 bp deletion in the *egt* gene (ctagagatctctagagatct), and a 1 bp SNP in the hr3 (A > G) (Table 5). Since the hrs have highly repetitive sequences, making these regions difficult to amplify and sequence, it is not conclusive whether the 1 bp SNP is a true mutation or a sequencing artifact. The FASTA file of the tiled-amplicon-sequenced p6.9GFP rBEV from the scrambled control can be accessed from Borealis, The Canadian Dataverse Repository (https://doi.org/10.5683/SP3/FIBEX4, accessed on 20 February 2025).

### 3.5. Lowering Infectious Budded Virus from Sf9-Cas9 Cells Through Plasmid-Based Delivery of sgRNA Targeting *gp64*

**Figure 4:**
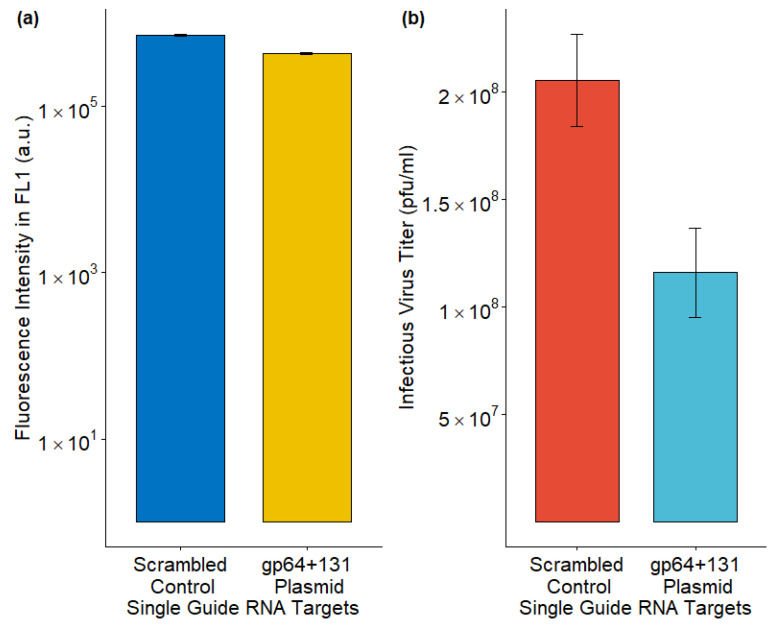
Effect on (**a**) foreign protein production and (**b**) infectious virus titer upon CRISPR-Cas9 based T-I assay. The data from three replicates are presented here.

The *Ac*MNPV major glycoprotein gene, *gp64*, was disrupted by the CRISPR-Cas9-based T-I assay [5]. While the fluorescence intensity, which represented GFP production, was similar to the control, the IVT was reduced upon *gp64* disruption (Figure 4).

The extracted gDNA of the p6.9GFP rBEV upon *gp64* gene disruption was sequenced by tiled-amplicon sequencing and compared to the shotgun-sequenced rBEV as the reference genome. The 35 and 20 bp deletions observed in the p6.9GFP rBEV genome from the scrambled control were also detected in the consensus sequence of the gp64+131 targeted p6.9GFP rBEV gDNA. Across the *gp64* gene, an average read depth of 156 was obtained (with a minimum read depth of 100) using the Pilon/DNAdiff pipeline. The Pilon/DNAdiff pipeline alone did not reveal any mutations within *gp64*; however, utilizing the CRISPResso2 tool, 23 reads were aligned with the provided *gp64* reference amplicon sequence, and 4 (17.39%) of those reads revealed indel mutations within the *gp64* target region. Table 6 shows the mutations observed within *gp64* for each of the four reads (alleles). The FASTA file of the targeted p6.9GFP rBEV and the detailed report generated by CRISPResso2 have been deposited in Borealis, The Canadian Dataverse Repository (https://doi.org/10.5683/SP3/FIBEX4, accessed on 20 February 2025).

## 4. Discussion

*Ac*MNPV is the most widely used baculovirus of the BEVS for the production of recombinant proteins, VLPs, vaccines, and other biologics [36,37,38]. While sequence data are available for the original *Ac*MNPV C6 [2] and E2 [34] strains, to the best of our knowledge, there is no information on the whole-genome sequence of the rBEVs constructed with an *Ac*MNPV DNA backbone and transfer plasmid. The major goal of this work was to generate the consensus sequence of the p6.9GFP rBEV and develop a pipeline to efficiently confirm the mutations upon CRISPR-Cas9 targeting. To achieve these tasks, two different sequencing methods were employed—shotgun sequencing and a tiled-amplicon sequencing assay. Although shotgun sequencing is fast and cost-effective compared to traditional approaches such as Sanger sequencing, it has limitations, including the requirement for a high concentration of starting material and difficulties in resolving repetitive regions, leading to assembly errors or gaps. On the other hand, the more specific tiled-amplicon sequencing assay with low DNA input requirements can encounter challenges, including dependence on a reference genome to design primers and the inability to amplify regions if mutations exist in primer-binding sites.

Baculoviruses with large dsDNA genomes have high mutation rates despite the known proofreading activity of its DNA polymerase [33,39,40]. A recent study was the first to report on the empirical estimates of mutation rates for *Ac*MNPV after five serial passages in insect larvae and estimated a mutation rate of 1 × 10^−7^ s/n/r (mutation rate per base per strand copying) when the most stringent criteria for mutation calling were applied [33]. In this study, we used shotgun sequencing to confirm the sequence of the p6.9GFP rBEV generated in our laboratory: an rBEV constructed utilizing an *Ac*MNPV DNA backbone derived from the C6 strain and a transfer plasmid carrying GFP under the p6.9 promoter [5]. This data is especially important when targeted genome editing is used to probe the rBEV genome. Our shotgun-sequenced rBEV consensus genome is 99.7% similar to the reference genome. The major differences with large indels between the reference genome and the shotgun-sequenced rBEV occurred mainly in the hrs, which are known to be prone to mutations [33,40].

### 4.1. Differences Compared to Reported Genomes from Shotgun Sequencing

Small indels and SNPs that were observed in genes/ORFs, such as *AcOrf-17*, *AcOrf-20*, *AcOrf-21*, *AcOrf-58*, *AcOrf-59*, *AcOrf-106*, *AcOrf-107*, *AcOrf-112*, and *pe/pp34*, within our shotgun-sequenced rBEV were also seen in the *Ac*MNPV WP10 (Wild Population 2010) consensus sequence compared to the *Ac*MNPV C6 genome [40]. Our shotgun data also revealed that only when the original ORFs, *AcOrf-20/21*, *58/59*, *106/107*, and *112/113* were combined into single ORFs, and the original ORFs, *AcOrf-17*, *AcOrf-52*, and *AcOrf-131* (*pe/pp34*) were stretched to a greater length, did stop codons appear at the appropriate locations. This is in line with reports that these ORFs should be combined and are not consistent with the original ORF classification [2,4,41].

A 40 bp insertion region in *egt*, consisting of four ten-base repeats, included a premature stop codon and resulted in a frameshift that caused multiple stop codons to be detected downstream of the insertion. It was previously reported that *egt* deletions commonly occurred upon baculovirus propagation in cell culture and are not essential for in vitro replication [42,43]. Other SNPs and small indels in our shotgun-sequenced rBEV are possibly uniquely related to the virus stock in question, emphasizing the need for whole-genome sequencing before targeted gene editing. Shotgun sequencing provided a consensus sequence for the p6.9GFP rBEV used in this study.

### 4.2. Tiled-Amplicon Sequencing to Assess CRISPR-Cas9 Editing

The CRISPR-Cas9 genome-editing technology has gained traction in the last decade. Targeted gene disruption in *Ac*MNPV rBEVs was achieved by co-transfection of *Ac*MNPV gDNA with Cas9/sgRNA RNP complexes and is the first report to establish the potential of CRISPR-Cas9 for baculovirus gene editing to improve the baculovirus as an expression vector and a biopesticide [44]. A more recent study focused on adapting the CRISPR-Cas9 technology to *Ac*MNPV/Sf9 cells and comparing gene disruption and transcriptional repression in *Ac*MNPV vectors via CRISPR-Cas9/dCas9 [16]. In another study, a sensitive CRISPR-Cas9-based transfection-infection assay capable of efficiently scrutinizing the *Ac*MNPV genome by targeted gene disruption was developed to aid optimization of the BEVS for improved biologics production [5]. Previous studies have portrayed that CRISPR-Cas9 mediated targeting of *gp64* reduced the co-production of baculovirus in cell cultures [17,18]. In our work, we observed that disruption of *gp64* by either infection of Sf9-Cas9 cells with the p6.9GFP_sgRNA_gp64+131 rBEV or transfection of Sf9-Cas9 cells with the gp64+131 plasmid followed by infection with the p6.9GFP rBEV resulted in significantly greater reduction in IVT compared to the effect on GFP production. These data confirmed that *gp64* gene disruption has the potential to minimize baculovirus contamination in cell cultures.

Although CRISPR-Cas9 can be effectively applied for targeted gene editing [5,17], changes in phenotypes alone cannot confirm the cut sites or any off-target effects. Some studies have utilized the previously described T7 endonuclease I assay to detect mutations only in the target regions [45,46]. Other studies confirmed CRISPR-Cas9 mutations by PCR-amplifying the target sites and ligating them into cloning vectors which are then sequenced by M13 primers [12,13]. However, these methods do not provide a comprehensive genome-wide analysis of any unintended effects. Whole-genome sequencing can compare the targeted genomes with the reference genome to screen for genome-wide off-targets and identify SNPs and indel mutations. In this work, we adapted the tiled-amplicon sequencing assay [19] to *Ac*MNPV vectors to sequence the whole rBEV genome upon CRISPR-Cas9 targeting. We postulated that this sequencing protocol could be widely utilized for rBEVs, and sequenced the p6.9GFP and the p6.9GFP_sgRNA_gp64+131 rBEV genomes from the multi-well plate experiments and recovered in the cell pellet.

#### 4.2.1. Sequencing Infected-Only and Scrambled Controls

A 100% coverage of the p6.9GFP rBEV genomes from the infected-only and scrambled controls was achieved with the tiled-amplicon primers generated for the reference genome (shotgun-sequenced rBEV) using PrimalScheme [19]. Compared to the reference genome, the p6.9GFP rBEV genomes from the two controls had a 35 bp deletion in one of the hrs (hr2). Previous studies have shown that deletion of individual hrs or combinations of up to 5 hrs does not significantly affect virus replication in cell cultures [47,48]. Thus, this 35 bp deletion in the hrs presumably does not affect rBEV replication. This deletion is believed to have not been detected by shotgun sequencing due to a reference genome assembly error. The tiled-amplicon sequencing assay, being more precise, was able to detect these deletions in the p6.9GFP rBEV genomes recovered from infected-only and scrambled controls. Additionally, the consensus sequence of the gp64+131 targeted p6.9GFP rBEV also had the same deletions seen in the controls, providing additional evidence that the deletion was only detected using the tiled-amplicon sequencing. The scrambled control differed from the infected-only control, with a shorter insertion in *egt*. Whereas the shotgun sequencing and the infected-only control showed a 40 bp insertion consisting of four ten-base repeats (5′-3′ ctagagatct), the scrambled control insertion consisted of only two of the same ten-base repeats. The consensus sequence of the gp64+131 targeted p6.9GFP rBEV also only had the 20 bp insertion of the same repeats.

#### 4.2.2. CRISPR-Cas9 Mutations Using p6.9GFP_sgRNA_gp64+131 rBEV in Sf9-Cas9 Cells

Phenotypically, the assay had a significant effect on the production of baculovirus when the p6.9GFP_sgRNA_gp64+131 rBEV was propagated in Sf9-Cas9 cells; however, as a result, the quantity of baculovirus in the supernatant was reduced, complicating viral gDNA recovery and sequencing. On the other hand, genomes recovered from the cell pellet underwent sequencing and revealed no mutations in *gp64* using Pilon/DNAdiff or CRISPResso2. It is not clear what happens to viral gDNA that does not undergo DNA repair once cleaved with a double-stranded break. The DNA damage response might signal the degradation of the viral genome via nucleases; however, we provide no evidence of this at this time. In this system, there is competition between the cleaving of the viral DNA and the replication and packaging of genomes. It is possible that the rate of replication is faster than the rate of targeting and cleaving of the viral genomes. The level of Cas9 in this system decreases with time post-infection because Cas9 is driven by an *Op*IE2 promoter, which is naturally downregulated as the infection progresses [16]. Furthermore, there will be a significant pool of genomes that the cell can use for homologous recombination, which could avoid mutations yet slow down the replication and packaging of viral genomes. In an attempt to find possible mutations, less stringent Trimmomatic parameters were used (SLIDINGWINDOW: 4:20, MINLEN: 170, LEADING: 15, TRAILING: 10) along with Pilon/DNAdiff on the sequencing data for both the reference and targeted p6.9GFP_sgRNA_gp64+131 rBEV genomes. The result of this analysis showed a duplication of the gene segments from *AcOrf-119* to *pe/pp34*, (which includes the *gp64* gene) in the assembled genomes. Within the first segment in p6.9GFP_sgRNA_gp64+131 rBEV, a large deletion (>50 bp) within the gp64+131 target region was found. Within the repeated region, and in p6.9GFP_sgRNA_gp64+131 rBEV, a large insertion (>50 bp) within the gp64+131 target region was observed. Although the duplication was seen in both the p6.9GFP_sgRNA_gp64+131 rBEV genome and the reference genome, the deletion and insertion were unique to the p6.9GFP_sgRNA_gp64+131 rBEV genome (propagated in the Sf9-Cas9 cells). Using the less stringent Trimmomatic parameters is not recommended, and should not be taken as hard evidence; however, in the absence of any other evidence, it points to the effect of the CRISPR-Cas9 editing.

#### 4.2.3. CRISPR-Cas9 Mutations Using a gp64+131 Plasmid in Sf9-Cas9 Cells with the T-I Assay

CRISPR-Cas9-mediated targeting can be utilized to identify genes that are not required for foreign protein production in cell culture and their disruption or removal can improve foreign protein yields [5]. Consistent with previous findings, using sgRNA plasmid transfection followed by infection yields a detectable effect that is not as large as what is observed if the sgRNA is provided by the baculovirus vector itself. This is understandable because uptake through transfection is not as efficient as with baculovirus vectors, nor do the plasmids replicate within the cell. It is therefore expected that some cells may not carry any sgRNA (even though 3.38 × 10^5^ plasmids per cell are used). Compared to CRISPR genomic editing of a single target within the cell, similar to the case of using the p6.9GFP_sgRNA_gp64+131 rBEV, we target a baculovirus gene while the virus is going through a productive infection cycle. All these factors combined make it difficult to investigate targeted genomes in a pool of viral gDNAs that either (1) bypassed CRISPR-mediated targeting by infecting cells that remained untransfected (no sgRNA); or (2) were repaired by homologous recombination using the untargeted genomes as a template. We still attempted to analyze CRISPR-mediated mutations within the targeted *gp64* gene using the CRISPResso2 pipeline. It is to be noted that our raw reads came from whole-genome sequencing of the rBEV and not just the regions flanking the target sequence. CRISPResso2 preprocessed 96.09% of the raw reads that passed the quality control. Overall, 82.09% of the reads also passed the CRISPResso2 filters (preprocessing) before being aligned with the reference amplicon. Since the tool was run from the Galaxy server, some parameters, such as the minimum alignment score (default: 60), were masked and could not be modified. The stringent filtering criteria applied by CRISPResso2 probably filtered out reads that could otherwise be aligned with the reference amplicon. This analysis resulted in a low abundance of reads (23 reads) that aligned with the *gp64* reference amplicon sequence, with deletions detected within the *gp64* target region for 4 (17.39%) of those reads. A detailed information file (info.pickle) and running log of this tool can be accessed via the CRISPResso2_p6.9GFP rBEV_sgRNAPlasmid_gp64 report, which can be obtained from Borealis.

### 4.3. Concluding Remarks

Our findings suggest that having a comprehensive knowledge of the sequence of the rBEV is essential to ensure specific sequences (genes) within its genome are being targeted, CRISPR-Cas9-mediated *gp64* gene disruption can be used as an effective tool to reduce budded virus contamination in cell cultures, and the bioinformatics pipeline can detect CRISPR-Cas9-mediated mutations within the target region. Nonetheless, the shotgun sequencing pipeline outlined here can be applied to generate a consensus sequence of other recombinant viruses for which only a best-guess sequence has been identified. Additionally, the tiled-amplicon sequencing assay pipeline can be applied to other viruses for which tiled-amplicon primers can be confidently designed; that is, the genome sequence is available. This sequencing assay being more specific and requiring a lower starting material concentration makes it easier to investigate the genomes of different viruses upon gene editing or amplification in cell cultures.

## Figures and Tables

**Table 1:** Multiplex PCR setup.

PCR Component	PCR Pool 1	PCR Pool 2
Q5^®^ High-Fidelity 2× Master Mix	12.5 µL	12.5 µL
Primer Pool 1 (10 µM)	2.5 µL	-
Primer Pool 2 (10 µM)	-	2.5 µL
DNA Template (1–10 ng) ^1^	10 µL	10 µL

^1^ Recovered rBEV gDNA used as the DNA template.

**Table 2:** Multiplex PCR amplification parameters.

PCR Step Number	PCR Step	Temperature	Duration
1	Initial denaturation	98 °C	30 s
2	Denaturation	95 °C	15 s
3	Annealing/extension	63 °C	5 min
N/A	Repeat steps 2 and 3 for 35 cycles	N/A	N/A
4	Hold	10 °C	Infinite

**Table 3:** Mutations from shotgun sequencing of p6.9GFP rBEV.

Mutation Region	Mutation Type	Mutation Length (bp)	*Ac*MNPV C6 Sequence	Shotgun Sequence	^a^ Amino Acid Change	Position on Shotgun Sequence
Homologous region 2 (hr2)	Deletion	49	ttaaact ^1^	-	Not applicable	27,333
Homologous region 2 (hr2)	Deletion	137	aactcgc ^2^	-	Not applicable	27,371
Homologous region 3 (hr3)	Deletion	72	aatcgtg ^3^	-	Not applicable	71,257
Homologous region 3 (hr3)	Deletion	72	caagtag ^4^	-	Not applicable	71,341
Homologous region 2 (hr2)	Insertion	1	-	C	Not applicable	27,341
Homologous region 2 (hr2)	SNPs	1	C	G	Not applicable	27,416
Homologous region 3 (hr3)	SNPs	1	A	C	Not applicable	71,255
Homologous region 3 (hr3)	SNPs	1	A	G	Not applicable	71,294
Homologous region 3 (hr3)	SNPs	1	A	G	Not applicable	71,306
Homologous region 3 (hr3)	SNPs	1	C	G	Not applicable	71,378
Homologous region 5 (hr5)	SNPs	1	T	C	Not applicable	115,201
Homologous region 5 (hr5)	SNPs	1	T	C	Not applicable	115,250
chiA/v-cath promoter	Deletion	37	tttaatt ^5^	-	Not applicable	105,599
AcOrf-106 promoter	Insertion	1	-	A	Not applicable	94,054
AcOrf-106 promoter	Insertion	1	-	A	Not applicable	94,124
AcOrf-106 promoter	Insertion	1	-	C	Not applicable	94,172
fgf promoter	SNPs	1	G	T	Not applicable	28,133
AcOrf-57 promoter	SNPs	1	A	T	Not applicable	47,546
AcOrf-78 promoter	SNPs	1	G	C	Not applicable	65,943
chiA/v-cath promoter	SNPs	7	taaaaaa	cccgggc	Not applicable	105,601
^‡^ lef2/AcOrf-603 recombination region	SNPs	2	CT	TC	Not applicable	3743
*pk-1*	Deletion	1	T	-	-	8157
*AcOrf-17*	Deletion	1	A	-	-	14,943
*AcOrf-21*	Deletion	1	C	-	-	17,024
*AcOrf-112*	Deletion	1	A	-	-	97,094
*ie-01*	Deletion	2	CG	-	-	122,767
*ie-01*	Deletion	1	A	-	-	123,928
*pk-1*	Insertion	1	-	C	His	8143
*egt*	Insertion	40	-	ctagaga ^6^	^†^ LEISRDL*RSLEIS	12,428
*AcOrf-20*	Insertion	3	-	cgg	Pro	16,922
*AcOrf-52*	Insertion	1	-	G	Ala	44,907
*AcOrf-59*	Insertion	1	-	A	Asn	48,451
*AcOrf-106*	Insertion	2	-	CA	ThrAsn	94,260
*AcOrf-106*	Insertion	1	-	G	Glu	94,293
*AcOrf-106*	Insertion	2	-	CG	Pro	94,343
*AcOrf-107*	Insertion	6	-	atttgg	IleTrp	94,422
*AcOrf-107*	Insertion	1	-	A	Arg	94,433
*pe/pp34*	Insertion	1	-	G	Thr	109,299
*AcOrf-1629*	SNPs	1	G	A	Phe	6371
*AcOrf-11*	SNPs	1	G	A	Asp	8685
*AcOrf-11*	SNPs	1	G	A	Trp	9434
*AcOrf-20*	SNPs	1	A	G	Leu	16,888
*AcOrf-20*	SNPs	2	GC	CA	Leu	16,925
*AcOrf-20*	SNPs	2	AG	GA	ProPro	16,930
*AcOrf-20*	SNPs	2	CG	GC	ProGlu	16,933
*env-prot*	SNPs	1	T	A	Val	21,007
*p47*	SNPs	1	A	C	Val	33,392
*gta*	SNPs	1	G	A	Asn	34,844
*gta*	SNPs	1	G	T	Phe	34,895
*odv-e66*	SNPs	1	C	G	Met	37,998
*odv-e66*	SNPs	1	T	C	Ser	38,304
*AcOrf-51*	SNPs	1	A	T	Phe	44,182
*AcOrf-53*	SNPs	1	C	T	Phe	45,551
*AcOrf-54*	SNPs	1	T	G	Val	46,633
*AcOrf-57*	SNPs	1	C	T	Leu	47,770
*AcOrf-58*	SNPs	1	T	G	Arg	48,249
*lef9*	SNPs	1	G	A	Asn	50,209
*lef9*	SNPs	1	G	T	Ser	50,314
*tlp*	SNPs	1	G	T	Glu	68,066
*AcOrf-84*	SNPs	1	C	A	Arg	71,492
*AcOrf-93*	SNPs	1	C	G	Glu	79,916
*helicase*	SNPs	1	G	C	Leu	81,217
*AcOrf-106*	SNPs	1	A	C	Asn	94,263
*AcOrf-114*	SNPs	1	C	G	Gln	98,860
*AcOrf-120*	SNPs	1	A	G	Asp	102,644
*AcOrf-120*	SNPs	1	A	G	Leu	102,664
*pk-2*	SNPs	1	T	A	Phe	103,878
*94k*	SNPs	2	TA	CC	GluVal	113,302
*p10*	SNPs	1	C	G	Ser	116,722
*p74*	SNPs	2	GT	TG	IleMet	116,950
*p74*	SNPs	1	A	G	Gln	118,441
*p74*	SNPs	1	A	T	Glu	118,509
*me53*	SNPs	1	A	G	Arg	120,034
*me53*	SNPs	1	A	G	Thr	120,036
*ie-0/ie-01*	SNPs	1	G	A	Gln	120,596
*ie-01*	SNPs	1	T	C	Ala	122,767
*ie-01*	SNPs	1	T	G	Ala	122,768
*pe38*	SNPs	1	G	C	Arg	131,047

^a^ Amino acid changes in the coding regions of the shotgun-sequenced rBEV. These are supposed changes, as amino acids are not codon-optimized. ^†^ The amino acid code LEISRDL*RSLEIS is LeuGluIleSerArgAspLeu**STOP**ArgSerLeuGluIleSer. ^‡^ The mutation is not within the coding region of *lef2* or *AcOrf-603*. Complete deletion and insertion mutation sequences greater than 7 bp are listed here: ^1^ ttaaactcgctttacgagtaaaattctacttgtaacgcatgatcaaggg; ^2^ aactcgctttacgagtagaattctacttgtaacgcacgcccaagggatgatgtcatttatttgtgcaaagctgatgtcatcttttgcacacgattataaacacaatcaaataatgactcatttgtttttcaaaactg; ^3^ aatcgtgcgttacaagtagaattctactcgtaaagcgagttcggttttgaaaaacaaatgacatcatttctt; ^4^ caagtagaattctactcgtaaagcgagtttagttttgaaaaacaaatgacatcatctcttgattatgtttta; ^5^ tttaatttatcttaattttaagttgtaattattttat; ^6^ ctagagatctctagagatctctagagatctctagagatct.

**Table 4:** Changes observed in the gDNA of p6.9GFP_sgRNA_gp64+131 rBEV upon propagation in Sf9-Cas9 cells via tiled-amplicon sequencing assay.

Mutation Region	Mutation Type	Mutation Length (bp)	Reference/Targeted Genome ^1^	Position on Targeted rBEV Sequence
Homologous region 2	SNPs	2	CC/AT	28,491
Homologous region 3	SNPs	1	C/G	72,827
AcOrf-20 promoter	SNPs	1	C/G	17,961
*AcOrf-1629*	SNPs	1	A/G	7427
*pk-1*	Deletion	1	T/-	9210
*pk-1*	Insertion	1	-/C	9195
*AcOrf-11*	SNPs	1	G/A	9737
*AcOrf-84*	SNPs	1	C/A	72,941

^1^ Changes in nucleotides between the p6.9GFP_sgRNA_gp64+131 rBEV reference genome (amplified in Sf9 cells) on the left and the p6.9GFP_sgRNA_gp64+131 rBEV targeted genome (amplified in Sf9-Cas9 cells) on the right.

**Table 5:** Changes in the gDNA of p6.9GFP rBEV obtained from tiled-amplicon sequencing upon T-I assay.

Mutation Region	Mutation Type	Mutation Length (bp)	*Ac*MNPV C6/Scrambled Sequence ^1^
Homologous region 2	Deletion	35	aaatgat ^2^/-
Homologous region 3	SNPs	1	A/G
*egt*	Deletion	20	ctagaga ^3^/-

^1^ Changes in nucleotides between the *Ac*MNPV C6 strain-based reference genome (shotgun-sequenced rBEV) on the left and the scrambled control on the right; ^2^ aaatgatgtcattggatgagtcatttgtttttcaa, the complete 35 bp deletion sequence observed in the hr2; ^3^ ctagagatctctagagatct, and the complete 20 bp deletion sequence observed in the *egt* gene.

**Table 6:** Mutations within the *gp64* gene from tiled-amplicon sequencing of p6.9GFP rBEV upon *gp64* gene disruption.

Read	Mutation Region	Mutation Type	Mutation Length (bp)	Reference/Targeted *gp64* Sequence	Position on Targeted gDNA
Read 1	*gp64*	Deletion	12	gtccttttgcag/-	108,669
Read 2	*gp64*	Deletion	2	CC/-	108,671
Read 3	*gp64*	Deletion	1	C/-	108,671
Read 4	*gp64*	Deletion	2	GT/-	108,669

## Data Availability

The datasets generated during and/or analyzed during the current study are available from the corresponding author on reasonable request. The original datasets can also be publicly accessed from Borealis (https://doi.org/10.5683/SP3/FIBEX4, accessed on 20 February 2025).

## Supplementary Materials

The following supporting information can be downloaded at https://www.mdpi.com/article/10.3390/v17030437/s1, Table S1: Primers used to construct the scrambled control plasmid [23,24]; Table S2: Positions of the tiled-amplicon primers in the p6.9GFP genome (shotgun-sequenced rBEV) as generated by Primal Scheme [19] and used in this study. Forward primers are denoted by ‘+’ or LEFT and reverse primers by ‘-’ or RIGHT; Table S3: Tiled-amplicon primers generated by Primal Scheme [19] and used in this study; Table S4: Custom primers with unique i7 and i5 index pairs for each sample, used to construct MiSeq DNA libraries; Figure S1: Visual representation of sequence alignments (Benchling). The first panel is the shotgun-sequenced reference genome (p6.9GFP rBEV_shotgun.fasta), the second panel is the p6.9GFP rBEV genome from the infected-only control (p6.9GFP rBEV_TiledAmplicon_Infected-onlyControl.fasta), and the third panel is the p6.9GFP rBEV genome from the scrambled control (p6.9GFP rBEV_TiledAmplicon_ScrambledControl.fasta). The red vertical lines in the grey bars represent the mutations compared to the reference genome.

## Abbreviations

The following abbreviations are used in this manuscript:

*Ac*MNPV	*Autographa californica* multiple nucleopolyhedrovirus
au	arbitrary units
*Bm*NPV	*Bombyx mori* nucleopolyhedrovirus
BEVS	baculovirus expression vector system
BLT	bead-linked transposomes
EPDA	end-point dilution assay
eYFP	enhanced yellow fluorescent protein
GFP	green fluorescent protein
HIV-1	human immunodeficiency virus type 1
hpi	hours post infection
hpt	hours post-transfection
hrs	homologous repeat regions
HT1	hybridization buffer
IVT	infectious virus titer
mAG	monomeric Azami Green
MOI	multiplicity of infection
NGS	next-generation sequencing
ORF	open reading frame
ori	origin of replication
PBS	phosphate-buffered saline
pfu/mL	plaque-forming units per mL
rBEV	recombinant baculovirus expression vector
SFM	serum-free media
sgRNA	single guide RNA
SNPs	single nucleotide polymorphisms
T-I assay	transfection-infection assay
VLP	virus-like particle
WP10	wild population 2010

## References

[1] Van Oers M.M., Pijlman G.P., Vlak J.M. (2015). Thirty years of baculovirus-insect cell protein expression: From dark horse to mainstream technology. J. Gen. Virol..

[2] Ayres M.D., Howard S.C., Kuzio J., Lopez-Ferber M., Possee R.D. (1994). The Complete DNA Sequence of Autographa californica Nuclear Polyhedrosis Virus. Virology.

[3] Miele S.A.B., Garavaglia M.J., Belaich M.N., Ghiringhelli P.D. (2011). Baculovirus: Molecular Insights on Their Diversity and Conservation. Int. J. Evol. Biol..

[4] Rohrmann G.F. (2019). The AcMNPV genome: Gene content, conservation, and function. Baculovirus Molecular Biology.

[5] Bruder M.R., Aucoin M.G. (2023). A sensitive assay for scrutiny of *Autographa californica* Mult. Nucleopolyhedrovirus Genes Using CRISPR-Cas9. Appl. Microbiol. Biotechnol..

[6] Hitchman R.B., Possee R.D., Crombie A.T., Chambers A., Ho K., Siaterli E., Lissina O., Sternard H., Novy R., Loomis K. (2010). Genetic modification of a baculovirus vector for increased expression in insect cells. Cell Biol. Toxicol..

[7] Hitchman R.B., Possee R.D., Siaterli E., Richards K.S., Clayton A.J., Bird L.E., Owens R.J., Carpentier D.C.J., King F.L., Danquah J.O. (2010). Improved expression of secreted and membrane-targeted proteins in insect cells. Biotechnol. Appl. Biochem..

[8] Pijlman G.P., Van Den Born E., Martens D.E., Vlak J.M. (2001). Autographa californica baculoviruses with large genomic deletions are rapidly generated in infected insect cells. Virology.

[9] George S., Jauhar A.M., Mackenzie J., Kie S., Aucoin M.G. (2015). Temporal Characterization of Protein Production Levels From Baculovirus Vectors Coding for GFP and RFP Genes Under Non-Conventional Promoter Control. Biotechnol. Bioeng..

[10] Bassett A.R., Tibbit C., Ponting C.P., Liu J.L. (2014). Mutagenesis and homologous recombination in Drosophila cell lines using CRISPR/Cas9. Biol. Open.

[11] Chang J., Wang R., Yu K., Zhang T., Chen X., Liu Y., Shi R., Wang X., Xia Q., Ma S. (2020). Genome-wide CRISPR screening reveals genes essential for cell viability and resistance to abiotic and biotic stresses in Bombyx mori. Genome Res..

[12] Dong Z., Chen T., Zhang J., Hu N., Cao M., Dong F., Jiang Y., Chen P., Lu C., Pan M. (2016). Establishment of a highly efficient virus-inducible CRISPR/Cas9 system in insect cells. Antivir. Res..

[13] Dong Z., Huang L., Dong F., Hu Z., Qin Q., Long J., Cao M., Chen P., Lu C., Pan M. (2018). Establishment of a baculovirus-inducible CRISPR/Cas9 system for antiviral research in transgenic silkworms. Appl. Microbiol. Biotechnol..

[14] Liu Y., Chen D., Zhang X., Chen S., Yang D., Tang L., Yang X., Wang Y., Luo X., Wang M. (2022). Construction of baculovirus-inducible CRISPR/Cas9 antiviral system targeting BmNPV in *Bombyx mori*. Viruses.

[15] Mabashi-Asazuma H., Jarvis D.L. (2017). CRISPR-Cas9 vectors for genome editing and host engineering in the baculovirus–insect cell system. Proc. Natl. Acad. Sci. USA.

[16] Bruder M.R., Walji S.D., Aucoin M.G. (2021). Comparison of CRISPR-Cas9 Tools for Transcriptional Repression and Gene Disruption in the BEVS. Viruses.

[17] Bruder M.R., Aucoin M.G. (2023). Evaluation of Virus-Free Manufacture of Recombinant Proteins Using CRISPR-Mediated Gene Disruption in Baculovirus-Infected Insect Cells. Vaccines.

[18] Hausjell C.S., Klausberger M., Ernst W., Grabherr R. (2023). Evaluation of an inducible knockout system in insect cells based on co-infection and CRISPR/Cas9. PLoS ONE.

[19] Quick J., Grubaugh N.D., Pullan S.T., Claro I.M., Smith A.D., Gangavarapu K., Oliveira G., Robles-Sikisaka R., Rogers T.F., Beutler N.A. (2017). Multiplex PCR method for MinION and Illumina sequencing of Zika and other virus genomes directly from clinical samples. Nat. Protoc..

[20] Reed L.J., Muench H. (1938). A simple method for estimating fifty per cent endpoints. Am. J. Hyg..

[21] O’Reilly D.R., Miller L.K., Luckow V.A. (1992). Baculovirus Expression Vectors: A Laboratory Manual.

[22] Chakraborty M., Powichrowski J., Bruder M.R., Nielsen L., Sung C., Boegel S.J., Aucoin M.G. (2024). Probing Baculovirus Vector Gene Essentiality for Foreign Gene Expression Using a CRISPR-Cas9 System. Methods Mol. Biol..

[23] Port F., Chen H.M., Lee T., Bullock S.L. (2014). Optimized CRISPR/Cas tools for efficient germline and somatic genome engineering in Drosophila. Proc. Natl. Acad. Sci. USA.

[24] Claudi B., Spröte P., Chirkova A., Personnic N., Zankl J., Schürmann N., Schmidt A., Bumann D. (2014). Phenotypic variation of salmonella in host tissues delays eradication by antimicrobial chemotherapy. Cell.

[25] Bolger A.M., Lohse M., Usadel B. (2014). Trimmomatic: A flexible trimmer for Illumina sequence data. Bioinformatics.

[26] Bushnell B., Rood J., Singer E. (2017). BBMerge—Accurate paired shotgun read merging via overlap. PLoS ONE.

[27] Danecek P., Bonfield J.K., Liddle J., Marshall J., Ohan V., Pollard M.O., Whitwham A., Keane T., McCarthy S.A., Davies R.M. (2021). Twelve years of SAMtools and BCFtools. GigaScience.

[28] Eren A.M., Kiefl E., Shaiber A., Veseli I., Miller S.E., Schechter M.S., Fink I., Pan J.N., Yousef M., Fogarty E.C. (2021). Community-led, integrated, reproducible multi-omics with anvi’o. Nat. Microbiol..

[29] Walker B.J., Abeel T., Shea T., Priest M., Abouelliel A., Sakthikumar S., Cuomo C.A., Zeng Q., Wortman J., Young S.K. (2014). Pilon: An integrated tool for comprehensive microbial variant detection and genome assembly improvement. PLoS ONE.

[30] Katoh K., Rozewicki J., Yamada K.D. (2019). MAFFT online service: Multiple sequence alignment, interactive sequence choice and visualization. Briefings Bioinform..

[31] Pinello L., Canver M.C., Hoban M.D., Orkin S.H., Kohn D.B., Bauer D.E., Yuan G.C. (2016). Analyzing CRISPR genome-editing experiments with CRISPResso. Nat. Biotechnol..

[32] Clement K., Rees H., Canver M.C., Gehrke J.M., Farouni R., Hsu J.Y., Cole M.A., Liu D.R., Joung J.K., Bauer D.E. (2019). CRISPResso2 provides accurate and rapid genome editing sequence analysis. Nat. Biotechnol..

[33] Boezen D., Ali G., Wang M., Wang X., van der Werf W., Vlak J.M., Zwart M.P. (2022). Empirical estimates of the mutation rate for an alphabaculovirus. PLoS Genet..

[34] Maghodia A.B., Jarvis D.L., Geisler C. (2014). Complete genome sequence of the Autographa californica multiple nucleopolyhedrovirus strain E2. Genome Announc..

[35] Labun K., Montague T.G., Krause M., Torres Cleuren Y.N., Tjeldnes H., Valen E. (2019). CHOPCHOP v3: Expanding the CRISPR web toolbox beyond genome editing. Nucleic Acids Res..

[36] Pushko P., Tumpey T.M., Bu F., Knell J., Robinson R., Smith G.E. (2005). Influenza virus-like particles comprised of the HA, NA, and M1 proteins of H9N2 influenza virus induce protective immune responses in BALB/c mice. Vaccine.

[37] Pushko P., Kort T., Nathan M., Pearce M.B., Smith G.E., Tumpey T.M. (2010). Recombinant H1N1 virus-like particle vaccine elicits protective immunity in ferrets against the 2009 pandemic H1N1 influenza virus. Vaccine.

[38] Aucoin M.G., Mena J.A., Kamen A.A. (2010). Bioprocessing of Baculovirus Vectors: A Review. Curr. Gene Ther..

[39] Tomalski M.D., Wu J.G., Miller L.K. (1988). The location, sequence, transcription, and regulation of a baculovirus DNA polymerase gene. Virology.

[40] Chateigner A., Bézier A., Labrousse C., Jiolle D., Barbe V., Herniou E.A. (2015). Ultra deep sequencing of a baculovirus population reveals widespread genomic variations. Viruses.

[41] Harrison R.L., Bonning B.C. (2003). Comparative analysis of the genomes of Rachiplusia ou and Autographa californica multiple nucleopolyhedroviruses. J. Gen. Virol..

[42] Kumar S., Miller L.K. (1987). Effects of serial passage of Autographa californica nuclear polyhedrosis virus in cell culture. Virus Res..

[43] Cory J., Clarke E., Brown M., Hails R., O’Reilly D. (2004). Microparasite manipulation of an insect: The influence of the egt gene on the interaction between a baculovirus and its lepidopteran host. Funct. Ecol..

[44] Pazmiño-Ibarra V., Mengual-Martí A., Targovnik A.M., Herrero S. (2019). Improvement of baculovirus as protein expression vector and as biopesticide by CRISPR/Cas9 editing. Biotechnol. Bioeng..

[45] Liu Y., Ma S., Wang X., Chang J., Gao J., Shi R., Zhang J., Lu W., Liu Y., Zhao P. (2014). Highly efficient multiplex targeted mutagenesis and genomic structure variation in Bombyx mori cells using CRISPR/Cas9. Insect Biochem. Mol. Biol..

[46] Xiang Z., Ye Q., Zhao Z., Wang N., Li J., Zou M., Lau C.H., Zhu H., Wang S., Ding Y. (2024). Development of a baculoviral CRISPR/Cas9 vector system for beta-2-microglobulin knockout in human pluripotent stem cells. Mol. Genet. Genom..

[47] Carstens E.B., Wu Y. (2007). No single homologous repeat region is essential for DNA replication of the baculovirus Autographa californica multiple nucleopolyhedrovirus. J. Gen. Virol..

[48] Bossert M., Carstens E.B. (2018). Sequential deletion of AcMNPV homologous regions leads to reductions in budded virus production and late protein expression. Virus Res..

