# Supplementary material for: Adapting Next-Generation Sequencing to in Process CRISPR-Cas9 Genome Editing of Recombinant AcMNPV Vectors: From Shotgun to Tiled-Amplicon Sequencing
Source: Viruses. 2025 Mar 18;17(3):437. doi: 10.3390/v17030437 (PMC11946314; doi:10.3390/v17030437)
Supplement: Supplementary file 1 [file viruses-17-00437-s001.zip › viruses-3445658-supplementary.pdf]

Supplementary Materials: Adapting next-generation sequencing to *in process* CRISPR-Cas9 genome editing of recombinant *Ac*MNPV vectors: From shotgun to tiled-amplicon sequencing

Madhuja Chakraborty [1], Lisa Nielsen [1,2], Delaney Nash [2], Jozef I. Nissimov [2], Trevor C. Charles [2] and Marc G. Aucoin [1,*]

Table S1. Primers used to construct the scrambled control plasmid.

Template	Primer Sequence (5′-3′)	Amplicon
SfU6 gBlock gene fragment	atctgcttagggttaggcgt<br>cggtggtcgagcacgaattt	SfU6 promoter fragment[1]
pCFD4-U6:1_U6:3tandemgRNAs [1]	tcgaccaccgcaccttgaagcgcatgaactgttttagagctagaaatagc<br>cacaccacaaatatactgtt	gRNA scaffold with scrambled spacer sequence and SfU6 homologous sequence[2]
Amplicon 1 + Amplicon 2	atctgcttagggttaggcgt<br>cacaccacaaatatactgtt	SfU6-sgRNA DNA fragment
SfU6-sgRNA DNA fragment	tagaaacgcaatctgcttagggttaggc[†]<br>aagcggaagacacaccacaaatatactgttgc[†]	SfU6-sgRNA insert
pBR322-TIMER [2]	ttgtggtgtgtcttccgcttcctcgctc[†]<br>ctaagcagattgcgtttctacaaactcttttg[†]	Backbone fragment containing ampR and ori

[†] Gibson primers with overhangs for constructing pSfU6-sgRNA scrambled control plasmid by Gibson assembly; [1] Amplicon 1; [2] Amplicon 2.

Table S2. Positions of the tiled-amplicon primers in the p6.9GFP genome (shotgun-sequenced rBEV) as generated by Primal Scheme [3] and used in this study. Forward primers are denoted by '+' or LEFT and reverse primers by '-' or RIGHT.

Genome	Start Position (bp)	End Position (bp)	Primer Name	Pool	Forward/Reverse Primer	Sequence (5′-3′)
p6.9GFP	215	237	p6.9GFP_NGS_1_LEFT	1	+	TGACATCATCCACTGATCGTGC
p6.9GFP	5168	5190	p6.9GFP_NGS_1_RIGHT	1	-	ATGTCGTAGAAGAAGCAGTCGC
p6.9GFP	4861	4883	p6.9GFP_NGS_2_LEFT	2	+	AAGATCAAGCTGTGCATGAGGG
p6.9GFP	9662	9684	p6.9GFP_NGS_2_RIGHT	2	-	GCTCCCCTCCTTGCGAAATTAT
p6.9GFP	9286	9308	p6.9GFP_NGS_3_LEFT	1	+	CGATTTGCTCCAACACTTCACG
p6.9GFP	14277	14299	p6.9GFP_NGS_3_RIGHT	1	-	GTGCTTGGCAAAACACGTACTC
p6.9GFP	13904	13926	p6.9GFP_NGS_4_LEFT	2	+	TTTGACACACACCATCACCTCC
p6.9GFP	18910	18932	p6.9GFP_NGS_4_RIGHT	2	-	ATGCCGTACTTTTATCCAGGCC
p6.9GFP	18464	18486	p6.9GFP_NGS_5_LEFT	1	+	GTCTCAATCAGTGGACGTGCAT
p6.9GFP	23598	23620	p6.9GFP_NGS_5_RIGHT	1	-	TCGGCGATCTTGTTAGCATACG
p6.9GFP	23081	23103	p6.9GFP_NGS_6_LEFT	2	+	ACAAGTATGTGGACGTGTGCTC
p6.9GFP	28095	28117	p6.9GFP_NGS_6_RIGHT	2	-	TATCGCTTGCTGGCACTTGTAG
p6.9GFP	27734	27756	p6.9GFP_NGS_7_LEFT	1	+	TCGTTCTTCAGTGCCACATACG
p6.9GFP	32781	32803	p6.9GFP_NGS_7_RIGHT	1	-	AACATGGATTACATACGCGGCA
p6.9GFP	32393	32415	p6.9GFP_NGS_8_LEFT	2	+	ATTGATTGATCTCGAGCCACCG
p6.9GFP	37376	37398	p6.9GFP_NGS_8_RIGHT	2	-	AGTAGCGGTTGTAGCATTGAGC
p6.9GFP	36843	36865	p6.9GFP_NGS_9_LEFT	1	+	CAACGACGACGACCCCTATTTT
p6.9GFP	41987	42009	p6.9GFP_NGS_9_RIGHT	1	-	ACTCTGTGGCGGTAAACAAGTC
p6.9GFP	41476	41498	p6.9GFP_NGS_10_LEFT	2	+	GCTGCGGTTAAACACACCTTTC
p6.9GFP	46513	46535	p6.9GFP_NGS_10_RIGHT	2	-	CAGCTGCCCAAATTCCTGACTA
p6.9GFP	46014	46036	p6.9GFP_NGS_11_LEFT	1	+	GCCGAGGCGAGAAAAACAATTC
p6.9GFP	51107	51129	p6.9GFP_NGS_11_RIGHT	1	-	ATGATATATGTCGGCGCCACAC
p6.9GFP	50809	50831	p6.9GFP_NGS_12_LEFT	2	+	GGCTAGAGATGTTGTTGCGTGA

Table S2 continued from previous page

Genome	Start Position (bp)	End Position (bp)	Primer Name	Pool	Forward/Reverse Primer	Sequence (5′-3′)
p6.9GFP	55567	55589	p6.9GFP_NGS_12_RIGHT	2	-	CATATTTCCGTCTTGCCGCAAC
p6.9GFP	55190	55212	p6.9GFP_NGS_13_LEFT	1	+	GATGCTTTCGACATGTTGTGGC
p6.9GFP	60024	60046	p6.9GFP_NGS_13_RIGHT	1	-	GCGGGTTCAACGACATGGTATA
p6.9GFP	59646	59668	p6.9GFP_NGS_14_LEFT	2	+	ACAACGAGAGAATAAGAGCGGC
p6.9GFP	64499	64521	p6.9GFP_NGS_14_RIGHT	2	-	TAATGAACCACGAATCCTCCGC
p6.9GFP	64211	64233	p6.9GFP_NGS_15_LEFT	1	+	GCCGGGCTGACGATAATAAACA
p6.9GFP	69071	69093	p6.9GFP_NGS_15_RIGHT	1	-	TCGTTAACTTTACACTGGCCGG
p6.9GFP	68778	68800	p6.9GFP_NGS_16_LEFT	2	+	CGAGTTTATTTTGAGCGGCGAC
p6.9GFP	73714	73736	p6.9GFP_NGS_16_RIGHT	2	-	CACAAAAATCAGAGCCGTGCTG
p6.9GFP	73308	73330	p6.9GFP_NGS_17_LEFT	1	+	CCCGAAATGCCTATCAACACCA
p6.9GFP	78317	78339	p6.9GFP_NGS_17_RIGHT	1	-	TGACTTTAACAGCTCGGACTGC
p6.9GFP	78016	78038	p6.9GFP_NGS_18_LEFT	2	+	CACCGCTACAGCAGAGCAATTA
p6.9GFP	82847	82869	p6.9GFP_NGS_18_RIGHT	2	-	GCCGACGAGCTCAGCATTTATA
p6.9GFP	82526	82548	p6.9GFP_NGS_19_LEFT	1	+	CCATCAAGCAGACTTTTAGCGC
p6.9GFP	87495	87517	p6.9GFP_NGS_19_RIGHT	1	-	GCGGTACCGAAATTCCGTTTTG
p6.9GFP	87140	87162	p6.9GFP_NGS_20_LEFT	2	+	ATGTGGTACCGGTTGAAGAACG
p6.9GFP	92156	92178	p6.9GFP_NGS_20_RIGHT	2	-	AAGCAACTGTGACGCCATAGAC
p6.9GFP	91767	91789	p6.9GFP_NGS_21_LEFT	1	+	CCAACAAACAGCCCAACATGAG
p6.9GFP	96662	96684	p6.9GFP_NGS_21_RIGHT	1	-	GCCTGTGCTGCACTATGGATAA
p6.9GFP	96138	96160	p6.9GFP_NGS_22_LEFT	2	+	GGTTCCACCAAATTGTGAGGGA
p6.9GFP	101172	101194	p6.9GFP_NGS_22_RIGHT	2	-	CGTGTGCATTGCCTTCGATTAC
p6.9GFP	100792	100814	p6.9GFP_NGS_23_LEFT	1	+	TGCCATTTGTCCGCAATTGTTT
p6.9GFP	105698	105720	p6.9GFP_NGS_23_RIGHT	1	-	AGGCGAGACTTGAACTCACAAC
p6.9GFP	105199	105221	p6.9GFP_NGS_24_LEFT	2	+	GTAACGGCCAATTCAACGTGAC
p6.9GFP	110209	110231	p6.9GFP_NGS_24_RIGHT	2	-	GGTCAACGACGCAAACATGATG
p6.9GFP	109698	109720	p6.9GFP_NGS_25_LEFT	1	+	GGGAGAGTGCCGTTTTTCAAGA

Table S2 continued from previous page

Genome	Start Position (bp)	End Position (bp)	Primer Name	Pool	Forward/Reverse Primer	Sequence (5′-3′)
p6.9GFP	114802	114824	p6.9GFP_NGS_25_RIGHT	1	-	CCGAATTTTTGAACGACGACGG
p6.9GFP	114292	114314	p6.9GFP_NGS_26_LEFT	2	+	TCGGGTCCTATACGAAGCGTTA
p6.9GFP	119276	119298	p6.9GFP_NGS_26_RIGHT	2	-	ATATAGTGTTGCAGCGCTACCG
p6.9GFP	118924	118949	p6.9GFP_NGS_27_LEFT	1	+	TTCGTTGTGCATTTCAAAGCTTTTG
p6.9GFP	123722	123744	p6.9GFP_NGS_27_RIGHT	1	-	TGGTAAAAAGCGAATGGTCCGT
p6.9GFP	123430	123452	p6.9GFP_NGS_28_LEFT	2	+	CGTTAAAATGCTAAGCCGCGAG
p6.9GFP	128206	128228	p6.9GFP_NGS_28_RIGHT	2	-	CAACTGAACCCGTCGTCTGATT
p6.9GFP	126523	126545	p6.9GFP_NGS_29_LEFT	1	+	TTCCGTTGTCCGACGCTATAAC
p6.9GFP	131340	131363	p6.9GFP_NGS_29_RIGHT	1	-	TTGGGGTCAACATCGATAGTGTC
p6.9GFP	129190	129212	p6.9GFP_NGS_30_LEFT	2	+	CAAACAAATCTGGCGACTGTGG
p6.9GFP	2409	2431	p6.9GFP_NGS_30_RIGHT	2	-	CGGTGTACAGATACTTGTGCGT

Table S3. Tiled-amplicon primers generated by Primal Scheme [3] and used in this study.

Primer Name	Pool	Sequence (5′-3′)	Size (bp)	%GC	Tm (use 65)
p6.9GFP_NGS_1_LEFT	1	TGACATCATCCACTGATCGTGC	22	50	60.92
p6.9GFP_NGS_1_RIGHT	1	ATGTCGTAGAAGAAGCAGTCGC	22	50	60.91
p6.9GFP_NGS_2_LEFT	2	AAGATCAAGCTGTGCATGAGGG	22	50	61.13
p6.9GFP_NGS_2_RIGHT	2	GCTCCCCTCCTTGCGAAATTAT	22	50	60.93
p6.9GFP_NGS_3_LEFT	1	CGATTTGCTCCAACACTTCACG	22	50	60.84
p6.9GFP_NGS_3_RIGHT	1	GTGCTTGGCAAAACACGTACTC	22	50	61.03
p6.9GFP_NGS_4_LEFT	2	TTTGACACACACCATCACCTCC	22	50	60.93
p6.9GFP_NGS_4_RIGHT	2	ATGCCGTACTTTTATCCAGGCC	22	50	60.93
p6.9GFP_NGS_5_LEFT	1	GTCTCAATCAGTGGACGTGCAT	22	50	61.11
p6.9GFP_NGS_5_RIGHT	1	TCGGCGATCTTGTTAGCATACG	22	50	61.03
p6.9GFP_NGS_6_LEFT	2	ACAAGTATGTGGACGTGTGCTC	22	50	61.05
p6.9GFP_NGS_6_RIGHT	2	TATCGCTTGCTGGCACTTGTAG	22	50	61.17
p6.9GFP_NGS_7_LEFT	1	TCGTTCTTCAGTGCCACATACG	22	50	61.1
p6.9GFP_NGS_7_RIGHT	1	AACATGGATTACATACGCGGCA	22	45.45	60.92
p6.9GFP_NGS_8_LEFT	2	ATTGATTGATCTCGAGCCACCG	22	50	60.98
p6.9GFP_NGS_8_RIGHT	2	AGTAGCGGTTGTAGCATTGAGC	22	50	61.17
p6.9GFP_NGS_9_LEFT	1	CAACGACGACGACCCCTATTTT	22	50	61.11
p6.9GFP_NGS_9_RIGHT	1	ACTCTGTGGCGGTAAACAAGTC	22	50	60.99
p6.9GFP_NGS_10_LEFT	2	GCTGCGGTTAAACACACCTTTC	22	50	61.03
p6.9GFP_NGS_10_RIGHT	2	CAGCTGCCCAAATTCCTGACTA	22	50	60.8
p6.9GFP_NGS_11_LEFT	1	GCCGAGGCGAGAAAAACAATTC	22	50	61.16
p6.9GFP_NGS_11_RIGHT	1	ATGATATATGTCGGCGCCACAC	22	50	61.11
p6.9GFP_NGS_12_LEFT	2	GGCTAGAGATGTTGTTGCGTGA	22	50	61.11
p6.9GFP_NGS_12_RIGHT	2	CATATTTCCGTCTTGCCGCAAC	22	50	60.97
p6.9GFP_NGS_13_LEFT	1	GATGCTTTCGACATGTTGTGGC	22	50	61.16

Table S3 continued from previous page

Primer Name	Pool	Sequence (5′-3′)	Size (bp)	%GC	Tm (use 65)
p6.9GFP_NGS_13_RIGHT	1	GCGGGTTCAACGACATGGTATA	22	50	60.92
p6.9GFP_NGS_14_LEFT	2	ACAACGAGAGAATAAGAGCGGC	22	50	60.91
p6.9GFP_NGS_14_RIGHT	2	TAATGAACCACGAATCCTCCGC	22	50	60.92
p6.9GFP_NGS_15_LEFT	1	GCCGGGCTGACGATAATAAACA	22	50	61.24
p6.9GFP_NGS_15_RIGHT	1	TCGTTAACTTTACACTGGCCGG	22	50	61.05
p6.9GFP_NGS_16_LEFT	2	CGAGTTTATTTTGAGCGGCGAC	22	50	60.96
p6.9GFP_NGS_16_RIGHT	2	CACAAAAATCAGAGCCGTGCTG	22	50	61.1
p6.9GFP_NGS_17_LEFT	1	CCCGAAATGCCTATCAACACCA	22	50	61.13
p6.9GFP_NGS_17_RIGHT	1	TGACTTTAACAGCTCGGACTGC	22	50	61.05
p6.9GFP_NGS_18_LEFT	2	CACCGCTACAGCAGAGCAATTA	22	50	61.17
p6.9GFP_NGS_18_RIGHT	2	GCCGACGAGCTCAGCATTTATA	22	50	61.04
p6.9GFP_NGS_19_LEFT	1	CCATCAAGCAGACTTTTAGCGC	22	50	60.65
p6.9GFP_NGS_19_RIGHT	1	GCGGTACCGAAATTCCGTTTTG	22	50	61.15
p6.9GFP_NGS_20_LEFT	2	ATGTGGTACCGGTTGAAGAACG	22	50	61.05
p6.9GFP_NGS_20_RIGHT	2	AAGCAACTGTGACGCCATAGAC	22	50	61.37
p6.9GFP_NGS_21_LEFT	1	CCAACAAACAGCCCAACATGAG	22	50	60.73
p6.9GFP_NGS_21_RIGHT	1	GCCTGTGCTGCACTATGGATAA	22	50	60.93
p6.9GFP_NGS_22_LEFT	2	GGTTCCACCAAATTGTGAGGGA	22	50	60.94
p6.9GFP_NGS_22_RIGHT	2	CGTGTGCATTGCCTTCGATTAC	22	50	60.96
p6.9GFP_NGS_23_LEFT	1	TGCCATTTGTCCGCAATTGTTT	22	40.91	60.67
p6.9GFP_NGS_23_RIGHT	1	AGGCGAGACTTGAACTCACAAC	22	50	60.98
p6.9GFP_NGS_24_LEFT	2	GTAACGGCCAATTCAACGTGAC	22	50	60.84
p6.9GFP_NGS_24_RIGHT	2	GGTCAACGACGCAAACATGATG	22	50	61.15
p6.9GFP_NGS_25_LEFT	1	GGGAGAGTGCCGTTTTTCAAGA	22	50	61.25
p6.9GFP_NGS_25_RIGHT	1	CCGAATTTTTGAACGACGACGG	22	50	61.14
p6.9GFP_NGS_26_LEFT	2	TCGGGTCCTATACGAAGCGTTA	22	50	60.93
p6.9GFP_NGS_26_RIGHT	2	ATATAGTGTTGCAGCGCTACCG	22	50	61.04

Table S3 continued from previous page

Primer Name	Pool	Sequence (5′-3′)	Size (bp)	%GC	Tm (use 65)
p6.9GFP_NGS_27_LEFT	1	TTCGTTGTGCATTTCAAAGCTTTTG	25	36	60.82
p6.9GFP_NGS_27_RIGHT	1	TGGTAAAAAGCGAATGGTCCGT	22	45.45	60.99
p6.9GFP_NGS_28_LEFT	2	CGTTAAAATGCTAAGCCGCGAG	22	50	61.02
p6.9GFP_NGS_28_RIGHT	2	CAACTGAACCCGTCGTCTGATT	22	50	61.05
p6.9GFP_NGS_29_LEFT	1	TTCCGTTGTCCGACGCTATAAC	22	50	60.91
p6.9GFP_NGS_29_RIGHT	1	TTGGGGTCAACATCGATAGTGTC	23	47.83	60.62
p6.9GFP_NGS_30_LEFT	2	CAAACAAATCTGGCGACTGTGG	22	50	60.78
p6.9GFP_NGS_30_RIGHT	2	CGGTGTACAGATACTTGTGCGT	22	50	60.85

Table S4. Custom primers with unique i7 and i5 index pairs for each sample, used to construct MiSeq DNA libraries.

Sample	i7 Primer ID	i7 Index (5′-3′)[†]	i5 Primer ID	i5 Index (5′-3′)[‡]
p6.9GFP_sgRNA_gp64+131 rBEV	N724	CGCTCAGT	S515	TTCTAGCT
p6.9GFP rBEV infected-only control	N721	CCTCTCTG	S517	AGAGTAGA
p6.9GFP rBEV gp64+131 plasmid	N714	TCATGAGC	S515	TTCTAGCT
p6.9GFP rBEV scrambled control	N707	GTAGAGGA	S517	AGAGTAGA

[†] All i7 indexes have the sequence CAAGCAGAAGACGGCATACGAGAT appended to their 5′ end and GTCTCGTGGGCTCGG to their 3′ end;
[‡] All i5 indexes have the sequence AATGATACGGCGACCACCGAGATCTACAC appended to their 5′ end and TCGTCGGCAGCGTC to their 3′ end.

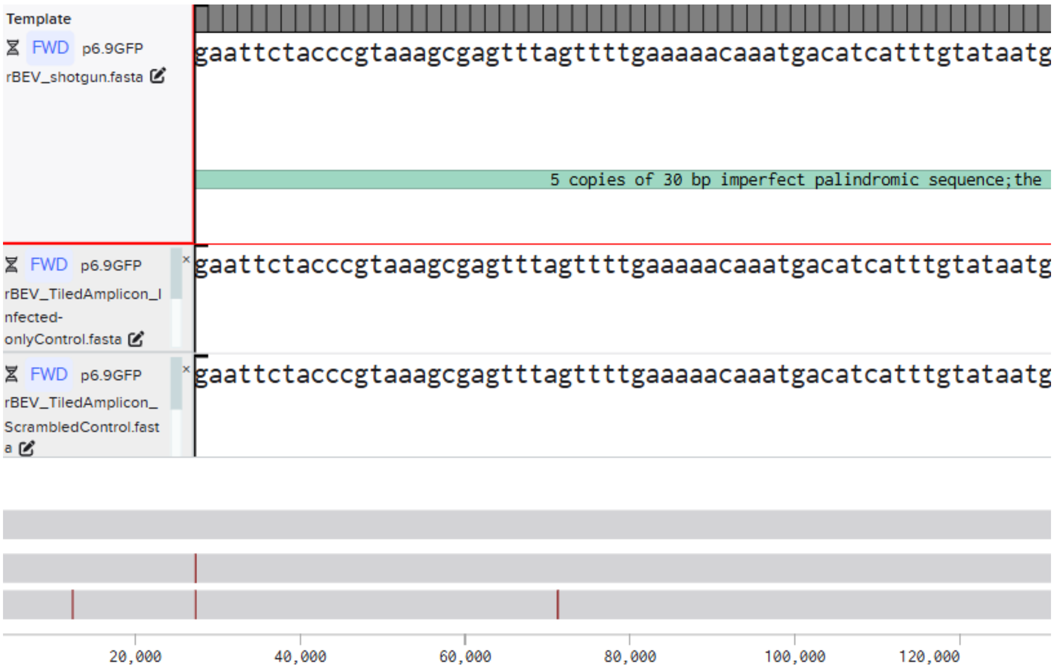

Figure S1. Visual representation of sequence alignments (Benchling). The first panel is the shotgun-sequenced reference genome (p6.9GFP rBEV_shotgun.fasta), the second panel is the p6.9GFP rBEV genome from the infected-only control (p6.9GFP rBEV_TiledAmplicon_Infected-onlyControl.fasta), and the third panel is the p6.9GFP rBEV genome from the scrambled control (p6.9GFP rBEV_TiledAmplicon_ScrambledControl.fasta). The red vertical lines in the grey bars represent the mutations compared to the reference genome.

References

1. Port, F.; Chen, H.M.; Lee, T.; Bullock, S.L. Optimized CRISPR/Cas tools for efficient germline and somatic genome engineering in Drosophila. *Proceedings of the National Academy of Sciences of the USA* **2014**, *111*.
2. Claudi, B.; Spröte, P.; Chirkova, A.; Personnic, N.; Zankl, J.; Schürmann, N.; Schmidt, A.; Bumann, D. Phenotypic variation of salmonella in host tissues delays eradication by antimicrobial chemotherapy. *Cell* **2014**, *158*, 722–733.
3. Quick, J.; Grubaugh, N.D.; Pullan, S.T.; Claro, I.M.; Smith, A.D.; Gangavarapu, K.; Oliveira, G.; Robles-Sikisaka, R.; Rogers, T.F.; Beutler, N.A.; et al. Multiplex PCR method for MinION and Illumina sequencing of Zika and other virus genomes directly from clinical samples. *Nature Protocols* **2017**, *12*, 1261–1276.

Disclaimer/Publisher's Note: The statements, opinions and data contained in all publications are solely those of the individual author(s) and contributor(s) and not of MDPI and/or the editor(s). MDPI and/or the editor(s) disclaim responsibility for any injury to people or property resulting from any ideas, methods, instructions or products referred to in the content.
